# Perceptions on evaluative and formative functions of external supervision of Rwandan primary healthcare facilities: A qualitative study

**DOI:** 10.1371/journal.pone.0189844

**Published:** 2018-02-20

**Authors:** Michael Schriver, Vincent Kalumire Cubaka, Sylvere Itangishaka, Laetitia Nyirazinyoye, Per Kallestrup

**Affiliations:** 1 Centre for Global Health, Department of Public Health, Aarhus University, Aarhus, Denmark; 2 Aarhus University Hospital, Aarhus, Denmark; 3 School of Medicine and Pharmacy, College of Medicine and Health Sciences, University of Rwanda, Kigali, Rwanda; 4 School of Public Health, College of Medicine and Health Sciences, University of Rwanda, Kigali, Rwanda; The Chinese University of Hong Kong, HONG KONG

## Abstract

**Background:**

External supervision of primary healthcare facilities in low- and middle-income countries often has a managerial main purpose in which the role of support for professional development is unclear.

**Aim:**

To explore how Rwandan primary healthcare supervisors and providers (supervisees) perceive evaluative and formative functions of external supervision.

**Design:**

Qualitative, exploratory study.

**Data:**

Focus group discussions: three with supervisors, three with providers, and one mixed (n = 31). Findings were discussed with individual and groups of supervisors and providers.

**Results:**

Evaluative activities occupied providers’ understanding of supervision, including checking, correcting, marking and performance-based financing. These were presented as sources of motivation, that in self-determination theory indicate introjected regulation. Supervisors preferred to highlight their role in formative supervision, which may mask their own and providers’ uncontested accounts that systematic performance evaluations predominated supervisors’ work. Providers strongly requested larger focus on formative and supportive functions, voiced as well by most supervisors. Impact of performance evaluation on motivation and professional development is discussed.

**Conclusion:**

While external supervisors intended to support providers’ professional development, our findings indicate serious problems with this in a context of frequent evaluations and performance marking. Separating the role of supporter and evaluator does not appear as the simple solution. If external supervision is to improve health care services, it is essential that supervisors and health centre managers are competent to support providers in a way that transparently accounts for various performance pressures. This includes delivery of proper formative supervision with useful feedback, maintaining an effective supervisory relationship, as well as ensuring providers are aware of the purpose and content of evaluative and formative supervision functions.

## Introduction

Supervision in healthcare is a context-dependent practice with multiple definitions, and is generally regarded a core part of assuring and improving quality of patient care [1–7]. Supervision may be distinguished based on its content of normative functions on one hand (*ensuring* standards of services), and formative and restorative functions on the other (*enabling* providers by supporting professional and personal development) [8].

In low-and middle income countries, managerial supervision of primary health care facilities is a common and often costly practice mainly characterised by visits of external supervisors from central or district level [9]. It is primarily oriented at ensuring performance, and often applies normative or administrative functions including an inspective, control-based supervisory approach [10–12], and to a lesser extent the formative and restorative such as joint problem solving and constructive feedback [9]. A Cochrane review was unable to determine the effect of managerial supervision in developing countries, and graded the quality of evidence as low or very low [12].

Contrariwise, internal supervision is conducted from within facilities and is often more oriented at formative and restorative functions [13]. Five reviews that related to supervision of primary healthcare facilities in developing countries sparsely or not at all described studies concerned with internal supervision [5,9,12,14,15].

Hierarchical supervision models in Africa have been suggested to stem from supervisory practices of an authoritarian colonial past [16]. Supportive supervision is described as the preferred alternative, transitioning from a traditional inspection-paradigm to emphasise formative functions for professional development of providers [13]. As such, supportive supervision may be seen as a reactive attempt to balance out a strictly normative supervision approach. Even for supportive forms of supervision the effect on quality of care in Africa is difficult to determine [14].

Descriptors of supervision (such as managerial, formative, supportive etc.) may represent a simplification of interrelated aspects. For instance, effective managerial supervision to *ensure* performance at a facility should include a focus on formative functions to *enable* competence development of providers, and vice-versa. The descriptors may show which aspects are emphasised.

Performance-based financing (PBF) is an increasing management practice in Africa, and often applies a system of external supervision to evaluate performance and determine reward size. In a systematic review, evidence on the value for money of PBF in low- and middle income countries was found weak [17]. Formative supervision has been described as a key ancillary component of PBF [18].

Combining evaluative and formative supervision functions under the role of one person or team may have conflicting outcomes, also in PBF [11,18]. For example, supervisees may refrain from disclosing their training needs in attempts to achieve positive evaluations [10]. Emphasising formative and restorative functions such as mentoring within an inherently disciplinary or normative supervision system may inadvertently mask inescapable challenges related to underlying power asymmetries and line management requirements [19]. In contrast, studies from the counselling domain show that supervisors who collaborate well with supervisees may handle a dual role constructively [20,21].

Using self-determination theory (SDT), it has been argued that PBF’s influence on performance depends on whether it allows satisfaction of basic psychological needs for relatedness (e.g. being valued by clients, colleagues, superiors etc.), competence (e.g. a feeling of doing well at work) and autonomy (e.g. having some degree of choice to work as one finds most effective) [22]. A PBF system that prevents satisfaction of these may harm overall performance by crowding out motivation congruent with a provider’s own values and preferences, even if it stimulates certain behaviours through rewards [22]. While some assume this is an inherent consequence of a performance orientated system [23], it has been argued that this depends on particular designs and implementations of PBF [22]. Some studies claim there is evidence financial rewards are harmless to intrinsic motivation, although they do not apply the nuanced conceptualisation of five motivation levels offered by SDT [24].

We found no studies from low-and-middle income countries on the relationship between ensuring (normative) and enabling (formative and restorative) functions of supervision.

### Context

In Rwanda, health centres (HCs) deal with more than 90% of all outpatient visits and contain nearly 60% of all beds in the entire health system [25]. These facilities serve as the first point of access to healthcare for most patients, and are run by nurses of whom the vast majority have a secondary school based nursing education known as an A2 degree [26]. External supervisors work from district hospitals and visit HCs regularly, i.e. monthly for some purposes [27].

There is supervision at multiple health system levels[28], and a reported lack of supportive supervision of district health services [29]. No official guidelines or publications were found with a complete or integrated account of the activities in external supervision of HCs. From written material external supervisors are expected to support professional development of primary care providers [29,30]. Also, they are required to conduct performance evaluations for PBF, which influences the size of funds allocated to health facilities and distributed as rewards to providers [27,31]. In one Rwandan study PHC providers felt scared by supervision and under suspicion by supervisors [32].

Rwanda’s PBF system has been found to increase activities with high payment rates and relatively few requirements for providers [31], and improve compliance with national and international norms of care [27]. Also, PBF in Rwanda was described as a guarantee of regular supervision visits to HCs [33]. In interviews with hospital-based Rwandan nurses, PBF was found unnecessary and personal and psychological support of staff insufficient [34,35], suggesting instead a system a system build on trust [35]. Also, district health managers did not highlight PBF as a driver of performance [36].

The aim of this study is to explore how providers and their external supervisors perceive performance-oriented evaluative functions and provider-oriented formative functions of external supervision of Rwandan HCs, and how these perceptions may agree or differ between providers and supervisors.

## Method

We apply a holistic perspective on external supervision, looking at both its ensuring and enabling aspects. A lack of studies on the system of external supervision in Rwanda led us to choose an exploratory and inductive approach, as reflected in our discussion topics (see “S1 Appendix”).

To design this study we used knowledge from informal observations from one researcher (VC) who formerly worked within the system of external supervision of HCs, and another (MS) who participated in several supervision visits as a guest. In these observations we found providers in HCs likely to obtain a shared supervision experience, as were also supervisors, who would arrive on visits as a team from the district hospital. We observed a relaxed and humorous atmosphere within these separate groups. Also, it is claimed that successful FGDs may produce broader and deeper understanding of an issue than in-depth interviews as they may more effectively stimulate memories, debate and disclosure [37]. These were main reasons to choose focus group discussions as our source of data, as further elaborated under the subheading Data. In “S2 Appendix” we describe the study using the COREQ criteria for reporting qualitative research.

### Sampling and recruitment

We included both supervisors and providers to obtain richer data, and potentially compare these groups. The number of HCs under a district hospital is the number of HCs a given supervision team is required to visit over a time period, no matter the number of supervisors. We purposively selected supervision teams in: 1) a district hospital among those with fewest attached HCs at the time of the study, 2) a district hospital among those with most, and 3) a district hospital with an average number of HCs. Our provider FGDs were done at the HC most recently visited by each of the 3 above-mentioned supervision teams. We excluded HC managers to reduce social desirability bias, and excluded providers who had not experienced supervision within 6 months to reduce recall bias. This gave 6 FGDs with a total of 15 supervisors and 16 primary healthcare providers (see Table 1). All selected facilities were part of the public health system and not beneficiaries of partnerships excessively enhancing their service quality compared to other public facilities in Rwanda.

**Table 1:** Participant characteristics.

Characteristics		FGD 1	FGD 2	FGD 3	FGD 4	FGD 5	FGD 6	Total
**No. of participants**		4	4	7	5	6	5	31
**Supervisors or providers**		Super-visors	Super-visors	Super-visors	Provi-ders	Provi-ders	Provi-ders	15 Supervisors 16 Providers
**Age: range**		30–46	32–56	29–50	31–42	27–41	29–40	27–56
**Gender**	**Male**	2	4	5	0	2	2	15
	**Female**	2	0	2	5	4	3	16
**Highest Degree**	**A2**	0	0	1	3	3	4	11
	**A1**	2	2	1	2	3	0	10
	**A0**	2	1	4	0	0	1	8
	**Other**	0	1	1	0	0	0	2
**Years since first health prof. graduation: range**		3–24	5–32	4–20	7–17	4–7	6–11	3–32
**No. who had clinical training in past year**		Yes: 1 No: 3	Yes: 1 No: 3	Yes: 2 No: 5	Yes: 0 No: 5	Yes: 0 No: 6	Yes: 4 No: 1	Yes: 8 No: 23
**Years as supervisor: range**		2–8	2–10	1–10				1–10

A2: Basic nursing certificate attained during secondary school. A1: Full nursing degree. A0: Bachelor degree.

Additionally, five participants who demonstrated notable skills in listening and critical thinking participated in a 7^th^ mixed FGD to discuss excerpts from previous FGDs that showed key problems around supervision. The mixed setup required supervisors and providers to listen to and take account of each others’ views in further discussing the problems, thereby further nuancing while also challenging the robustness of the findings. In this mixed FGD we put together three providers and two supervisors from separate districts to avoid influencing or harming existing supervisory relationships.

### Data

We conducted FGDs in separate groups of supervisor and provider teams as we hypothesised such could:

- Take advantage of existing collegial bonds among peer everyday co-workers to elicit discussions (no need for participants to get to know each other first).
- Allow experiences and claims to be immediately challenged by colleagues with a shared or similar experience
- Stimulate participants to remember and reflect around own supervision experiences through hearing those of colleagues.

A pilot FGD had more characteristics of a group interview than a discussion among participants and was thus excluded. This, together with literature on low-moderator-involvement in FGDs [38–40], helped us develop an approach to maximise chances of authentic group discussions and minimize reactivity towards researchers, characterised by the following:

- All discussions and presented text were in the local language Kinyarwanda, as preferred by all participants.
- A local, experienced social scientist (SI) moderated all FGDs. His main role was to facilitate a safe and comfortable atmosphere. He would mainly stay passive and silent, only contributing to steer back deviated discussions, clarify topics if needed or probe relevant discussions if needed.
- Participants were immediately activated by taking turns to read aloud information about the study.
- Discussion topics were given as a short sentence on a chart. Each new discussion topic (see “S1 Appendix”) was read aloud by another participant prior to discussing.
- Two other researchers were silently present in the far background to listen, observe non-verbal interaction, keep a discussion logbook and take notes.

After each FGD, three researchers (VC, SI, MS) discussed observations and FGD content. Some FGDs were more self-propelled than others, though all were found with authentic discussions among participants. Few participants were markedly silent, but were stimulated to talk by other participants, the moderator or when their turn came to read a theme. The 8 discussion topics began with general aspects before becoming more specific (see “S1 Appendix”).

FGDs were conducted Oct. 2013 to Jan. 2014. Their effective duration was minimum 58min, maximum 1h42min, average 1h26min. Additionally, the mixed FGD was 2h20min, making the total recording 10h56min. FGDs were transcribed verbatim, anonymized, translated into English by an external professional interpreter, and the FGD moderator control-checked by comparing audio to transcripts and transcripts to translations line by line. Markers for wordless sounds like laughs and non-verbal events like pauses were included.

### Analysis

Four researchers familiarised themselves with all transcripts [41] and inductively coded one FGD at a time to subsequently discuss and agree on a code index for each FGD. Coders harmonised these into one overall code index, which was subsequently applied to all transcripts by 2 coders (VC and MS) using the software MAXQDA 11. Intercoder agreement was compared paragraph by paragraph, and a final harmonized version was completed. The purpose of this process was to facilitate discussions and analysis of the material. In comparing supervisors and providers we focussed on perceptions that were not unique to single participants, but re-appeared among several supervisors or providers.

We were continuously in contact with participating supervisors and providers as well as other informants to clarify our emerging questions and discuss our hypotheses. We presented our findings in meetings for feedback and discussion: in March 2016 for 7 supervisors (of whom 3 participated in the study), and in October 2016 for 6 supervisors and another meeting for 8 providers (both in regions that had not participated in the study). This mainly confirmed our findings, and further refined our analysis. The manuscript was shared with an English-speaking supervisor participant prior to submitting.

### Ethical considerations

Ethical and research clearance for this study was granted by the Faculty of Medicine Research Ethics Committee at the National University of Rwanda (Review Approval Notice N0 15/FoMREC /2013). Signed informed consent was obtained from all study participants prior to participation.

## Results

Table 1 shows key characteristics of participants as answered in a questionnaire. Supervisors were mainly males (11/15), 30 years or above except for one. Almost all had received their latest degree more than 4 years ago. Seven had less than 3 years of experience as a supervisor, and one had more than 10 years experience. Nine had not received clinical training in the past year.

Providers were mostly female (12/16), three less than 30 years old. All had received their most recent degree more than 4 years ago. Only 1 had the advanced A0 bachelor degree, 5 had an A1 (full nursing) degree, and 10 had only the secondary school based nursing certificate called A2. Eleven had not received clinical training within the past 1 year.

With our focus on the relationship between evaluative and formative supervision functions, we found 4 related but analytically separable conceptual levels within which this was discussed: 1) perceived theoretical reasoning behind supervision, i.e. intended supervision purposes and desired outcomes, 2) formal categories of supervision, i.e. how supervision is perceived and requested to be formally structured, 3) experienced events during supervision (supervision activities), and 4) how supervision motivates supervisees (motivation effect). We present results salient to our research focus under these 4 conceptual levels, beginning with supervision purpose.

### Supervision purpose

Improved quality of care emerged as the main goal of or philosophy behind supervision in all FGDs. Further, almost all supervisors would speak of an aim that providers learn, often by using the concept “training” to describe the supervision purpose. Learning was rarely discussed as a supervision purpose among providers.

Performance evaluation or detection of weaknesses were frequently mentioned by supervisors as a supervision purpose, such as in FGD3:

The main aim is to evaluate the performances of health workers.

And in FGD2:

We do evaluation and find out that people have weaknesses in a given area.

Provider discussions rather described correcting mistakes as a supervision purpose, here by an A1 nurse in FGD5:

The main objective of supervision, it seems to me that it is inspection about the way work is done at health centers. In case there are some areas where work is done badly, then it is corrected.

Correcting was generally perceived as desirable, though a problem if done through reprimanding.

There were several indications that evaluative and formative purposes of supervision could point in opposite directions, as this dialogue in FGD3 between three supervisors discussing how the purposes collide when training and evaluation are combined during a visit:

Normally, you train a person to lift her/him out of the hole in which s/he has fallen, to help her/him reach somewhere; but when you lift and put her/him down in the hole at the same time, what have you done at that time? That is evaluation! [Laughs] (…). Because where you have lifted me, you have also put me down from the hill on which you have helped me because of lower marks you have given me. In that case, you notice that the supervision we do today is not formative as it should be.

(…) *When you evaluate there comes the marking*. *As a result*, *s/he forgets what you have told him/her because s/he is not happy*.

(…) You have given those recommendations (…). It is formative. Then, there can be an evaluation. It is like a punishment, really it upsets everyone.

While some differences were found between supervisors’ and providers’ ways of describing the purpose of supervision, we saw no indications of conceptual differences in the two groups’ understanding of desired supervision outcomes.

### Supervision categories

Table 2 is based on supervisor FGDs, and confirmed in subsequent informal conversations, previous observations and reading accessible guidelines within some supervision services. It shows 2 distinct categories of external supervision of HCs in Rwanda.

**Table 2:** Evaluative and formative (technical) supervision of health centres in Rwanda.

Category	Evaluative supervision	Formative/technical supervision
**Main objective**	Evaluate patient utilisation (*Quantité*) and quality aspects (*Qualité*) of selected services	Help providers improve performance
**Tools**	Complete checklists	Summary checklists and clinical guides
**Marks for PBF**	Yes	No
**Schedule**	*Quantité*: Monthly. Date pre-announced. *Qualité*: Quarterly. Date unannounced.	Quarterly, or as needed. Date pre-announced.

PBF: Performance Based Financing; Qualité and Quantité: Local French terms for two aspects of evaluative supervision.

External supervisors provide *evaluative supervision* (consisting of so-called quantité and qualité) aiming to ensure a certain service standard and applying performance evaluations for PBF. In addition, the same group of supervisors are responsible for providing *formative (technical) supervision* aiming to help healthcare providers improve skills and performance. The latter ideally takes place at a date prior to evaluation visits, but may in practice be combined with it.

Providers were likely to use the term *supervision* in its global sense for both evaluative and formative categories. In fact, most providers did not differ between the two categories of supervision, and the distinction was unknown to some, such as an experienced A1 nurse in FGD5:

I do not understand it. Are there “formative” and “evaluative” supervisions? Are they different?

On the contrary, supervisors in all discussions would differ between the ‘formative’ and ‘evaluative’ categories, usually referred to respectively as simply *supervision* and *evaluation*.

While one provider and few supervisors seemed satisfied with supervision as it is, many supervisors and providers suggested the formal supervision structure should give higher priority to formative functions, here an A2 nurse in FGD6:

Formative supervisions are not [happening] enough. Most of the time they come to give marks or count what is related to the money of PBF.

And an A2 nurse in the mixed FGD:

They [supervisors] give you marks (…). They mainly look at what you have done wrongly. They may even insult you; but teaching is not often seen.

No supervisor directly expressed appreciation for the task of evaluation. Some stated a preference for supportive functions, such as a supervisor in FGD3:

I like doing a supervision which is related to training [formative] rather than evaluating (…). This is because it makes me feel at peace that I have done my work as it is supposed to be.

Many supervisors described their line of work as pre-dominantly formative, either by using evaluation visits to provide feedback, or because visits in practice often combined functions of evaluative and formative supervision. Not all supervisors agreed whether this is the case on the ground, as this dialogue in FGD3 between two supervisors:

Though you are going to give her/him marks, it is the training aspect (…) which takes priority.

(…) It does not happen like that (…). Though we say that supervision aims at training (…), most of the time supervision is confused with evaluation in such a way that trainings are few.

A few times it was indicated that emphasis on evaluation tasks is a recent development, such as this supervisor in FGD1:

Nowadays, when we go for supervision [formative], we also go with a plan of evaluating them (…). This is not how it should be done because formerly training used to take place first and then you could come back to evaluate.

Some supervisors described PBF-accountability to dictate an overemphasis on evaluative compared to formative supervision, such as this supervisor in FGD1:

We dedicate more time for the evaluation, for we will also be held accountable for that to our superiors from the ministry of health during what is called PBF.

Providers would often express that supervisor visits would not contain the support needed, as this dialogue in FGD6 between two A2 nurses:

S/he leaves without explaining it to you [training], and then next time when s/he comes back (…) the same thing. As a result, you only remain in a routine.

Only waiting for the evaluation! And you even start inventing what you do not know.

The request for more focus on supportive and formative functions was uncontested, substantial and general. All supervisor FGDs described how or why providers tend to perceive them as evaluators rather than advisors/supporters, as a supervisor in FGD1:

Oftentimes the HC’s personnel take it [technical supervision] in the wrong way: they take it as an evaluation, in view of the fact that they are used to receiving an evaluation.

HC managers were also seen to put emphasis on evaluation and PBF, as explained by a supervisor in FGD3:

When you look at the heads of health centres, you really notice (…) s/he bears in mind that (…) you will give her/him money. It is there s/he perceives her own interests in you.

In FGD2 and FGD3 supervisors suggested that evaluators be different from the people providing formative supervision. This view was contested by supervisors who found evaluators should also lead formative sessions enabling them to bear these in mind during evaluation, and also found the dual role practical due to resource constraints. In the mixed FGD a provider expressed unease with the idea of being evaluated by someone she had not collaborated with for formative purposes.

### Activities in supervision

Several actions and activities were described to take place as part of supervision. We present the most prevalent of relevance to our focus.

Although our discussion topics did not directly probe “marks”, marking was spontaneously mentioned in all discussions and in almost all discussion topics, the majority among providers. Mostly problematic issues were discussed, many referring to the supervisory relationship. Several paragraphs showed discomfort among providers with marking, often as fear. In FGD6 an A0 nurse found it threatening:

If it [supervision] comes in a form of threatening, only giving marks, people will certainly be (…) telling lies.

Supervisors discussed discomfort as something inherent to the activities of supervision, where even honest feedback can feel uncomfortable, as mentioned by a supervisor in FGD3:

A person [provider] considers you as an enemy because you have told and shown her/him the truth. There are people whom the truth hurt in their ears.

And here a supervisor in FGD2 about supervisors’ experience with being supervised themselves:

Even if he [national supervisor] comes to give us some advice, we first feel discomfort (…). That fear is in the human nature.

It was often indicated and always undisputed that evaluation visits are ideally supportive, such as by including training and feedback. Supervisors readily described the content of constructive feedback or that evaluation visits should be preceded by formative supervision visits. However, several supervisors and most providers found this not to be the case.

There was general agreement that formative supervision visits should be planned and announced well in advance. Dates of evaluation visits (qualité, see Table 2) are intentionally unannounced, which was sometimes problematized. In FGD4 an experienced A2 nurse expressed that unannounced visits may be stressful:

As the years pass (…) I do not feel anything special when they say there is supervision. (…). Unless you are not aware of it [beforehand]; this is when you do not feel comfortable.

An A0 nurse argued in FGD6 that announcing the date of evaluation visits would allow providers to prepare, coordinate and align procedures:

When you know that at a certain date they may come for supervision [evaluation], you can start counting what you have done on a daily basis, and you document them very well.

Other providers felt too much time would be used for preparation if dates were announced, such as this A1 nurse in FGD5:

If they gave notification, we would not work; instead, we would be preparing ourselves.

Absenteeism is another problem for unannounced visits, and may lead to waste of time, as mentioned by a supervisor in FGD1

Normally we should go [for evaluation] without making prior notice (…). But it is a problem to go and find that the one you want to supervise is not around.

Table 3 shows advantages and disadvantages of unannounced evaluation visits emerging in our material.

**Table 3:** Advantages and disadvantages of unannounced evaluations.

	Advantages	Disadvantages
**Date NOT announced**	More realistic example of everyday performance	Fear and stress of regular surprise evaluations Difficult to learn under stress Wasteful if key providers are absent Providers may experience being under suspicion Intrinsic motivation may decrease
**Date announced**	Providers ready for evaluation stress Likely a better uptake of formative content Provider learn by preparing for evaluation	”Over-preparation” at expense of non-evaluated services Larger potential for biased evaluations

Neither providers nor supervisors discussed involvement of providers in defining training needs. And only supervisors discussed identification of training needs as part of supervision activities, for instance a supervisor in FGD1:

It [supervision overall] helps us to know both the level of each employee and which training s/he needs.

Supervisors discussed correcting of mistakes as one of several activities to support providers during supervision. In FGD2 a supervisor related it to joint decision-making:

We must go and talk to those in charge in a bit to correct them (…). You tell them “There is a problem” (…) you discuss and then you agree and decide together.

Supervisors use checklists to mark the tasks and services delivered by providers. Through PBF, service marks may lead to rewards to the facility. Still, providers consistently talked about marks as individual, using phrases like “I get…” or “they give me…”. There is often a perception of collective punishment, as this A1 nurse described in the mixed FGD:

The marks s/he [supervisor] has given you will affect the whole institution. Therefore, let me say the climate between you and her/him is not good because you always suspect each other.

And an A2 nurse in FGD6:

One service can cause the reduction of the money a health center may get.

This leaves a potential for internal blame for salary decrease or low marks (marks in one service may depend on marks in another), as indicated by these two A2 nurses in a dialogue in FGD6:

If they [supervisors] find the in-charge [of the stock] has not filled the forms, they give me zero (…). Yet, I am not responsible for managing the stock.

(…) S/he [in-charge of stock] should be the one to be punished.

Several providers seemed to lack basic knowledge about how performance is evaluated or requested more information about marks, checklists and how salary is influenced. In FGD4 an A2 nurse with more than 10 years experience was not aware all evaluations influence PBF:

So, is there also money from the quality supervision? From the marks/score they give us in that of quality? [Another participant: They add it to that of PBF]. I didn’t know that.

Neither supervisors nor providers discussed restorative aspects of supervision, such as support in personal or emotional matters relating to work.

### Provider motivation through supervision

There was an uncontested sense of appreciation for supervision among providers, and for several reasons. Knowledge development was mentioned in all FGDs such as in this dialogue in FGD4 between an A2 nurse followed by two A1 nurses:

Supervision is helpful for us.

*(…) It increases the knowledge of a supervised person*.

(…) This helps to improve the further performance (…). In fact, no one can work without [supervision]… it is said that no one is perfect.

There was a sense that supervision urges providers to perform better, such as by an A2 nurse in FGD5:

Normally when you are aware that someone will come to supervise what you are doing (…), this makes you do things with eagerness everyday.

And an A2 nurse in FGD4:

They [supervisors] can find out a mistake you have made unawares and then they give you like 80% marks. And then, you make an effort to get 100% marks.

Some providers proposed building motivation ‘in spite of’ as opposed to ‘because of’ PBF, as this A0 nurse in FGD6 reacting to a system perceived as driven by marks:

Let us not stick on the marks (…). We can follow the internal supervision and improve services. Then, even if s/he [supervisor] may come and give us zero, we keep providing quality services. Whoever comes to our service appreciates the service.

An extrinsic motivation source was demonstrated in all provider FGDs, for a few providers specifically about monetary reward, but mostly about high marks, as this A2 nurse in FGD4 indicating contingency of work satisfaction on marks:

They may give you 100% marks (…). You tell yourself “I am a worker” and then you are proud of your success.

There is also a motivation to avoid blame from supervisors, here from an A2 nurse in FGD6:

No body likes being blamed! Do you know how bad it is being blamed and shown corrections but stay doing the same thing.

Supervisors would talk of motivation as giving encouragement to providers, such as to prevent professional isolation as this supervisor in FGD1:

Another main objective of supervision is like encouragement. I think that if they [providers] stay at their work places always alone, they would feel lonely. But when they see people coming to teach them, they gain.

Marks are mainly seen as encouragement both by providers and supervisors; here a supervisor in FGD3:

When s/he gets good marks, PBF goes well, and s/he gets encouraged and offers good services.

Providers spoke of low marks as something feared, often making them want to hide from supervision. Sometimes it was described as a motivation to gain higher marks next time. The motivation from marks and PBF was sometimes questioned, including how it combines with teaching, as by a supervisor in the mixed FGD:

Those marks affect motivation. That is where the problem is. (…) I do not know whether those who supervise [formatively] should not be the ones to evaluate [for PBF]; I do not know.

Providers’ interest in supervision seemed contingent with delivery of PBF-rewards, as this supervisor explains in FGD1:

Sometimes you arrive there and you find that the employees are unhappy, complaining that they don’t even receive the PBF, and then (…) they don’t care about you and the supervision.

The interest could also depend on whether a required change is perceived as relevant by the provider, as mentioned by another supervisor in FGD1:

Due to the fact that s/he has got a routine of doing that [a procedure] very quickly without applying the norms [guidelines], s/he just feels that you are delaying him/her. (…) s/he thinks that you are wasting his/her time.

## Discussion

Within the 4 conceptual levels presented (purpose, categories, activities and provider motivation), providers appear similar as a group, as do supervisors. Table 4 gives a crude comparison of providers and supervisors based on these levels.

**Table 4:** Comparison of supervisors’ and providers’ main perceptions of supervision on four conceptual levels.

Perceived main…	Providers	Supervisors	Difference
**…1) purpose of supervision**	Improve care	Improve care	Negligible
**…2) supervision category**	Evaluative	Formative	Substantial
**…3) activities in supervision**	Pressures to perform (marking, correcting, suspicions, blame, surprise visits)	Efforts to support (useful feedback, joint problem solving, mutual learning)	Substantial
**…4) effect on provider motivation**	“Urges us to improve, but…”	“Helps them improve, but…”	Negligible

Table 4 shows that providers perceived supervision as structured around the category of evaluation visits, applying activities to control their performance through events such as unannounced visits, marking and correcting. We use the term “pressures to perform” to summarize these activities; not as a derogatory. The term rather reflects a performance-centered supervision system perceived to operate through a power of risks, in our data mainly the risk of low performance marks or blame. This was substantially different from the common supervisor perception of formative visits as the main supervision category, including activities such as provision of feedback, discussing problems and solutions with providers, mutual learning etc.

It is notable that providers found performance pressures motivating as they urged them to improve marks, be on their toes and fight idleness. However, the “but” in Table 4 (bottom row) represents providers’ strong request for a better relationship and collaboration with supervisors as well as a more formative supervision approach.

Paradoxically, supervisors too requested more focus on formative compared to evaluative functions, as represented with another “but” in Table 4, appearing to contradict their view that formative supervision was already the main operating category. It is also reflected in that all supervisor FGDs had disagreeing paragraphs on whether formative or evaluative activities dominated. This indicates a supervisor narrative that emphasises preferred tasks, or a perception that evaluation is structurally given by the PBF system, in which supervisors prefer to consider or present their own efforts as attempts to alleviate negative or strengthen positive outcomes of this system. This may mask underlying performance pressures, as well as point to a “fire-fighting identity” among some supervisors, as they must deal with challenges or conflicts arising from performance pressures in PBF.

### Determinants in supervision

**Fig 1:**
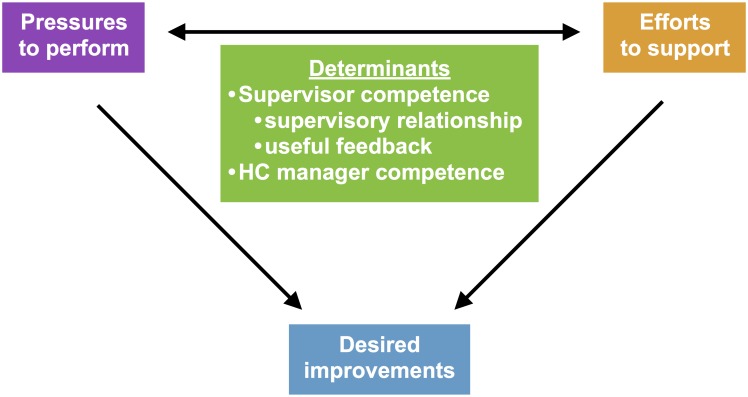
Determinants of the outcome of pressures to perform and efforts to support.

While our study shows that evaluation practices easily conflict with those of support, it also indicates that this conflict may be alleviated. The competences of the supervisor and the HC manager are two major factors (Fig 1) that in our data appear to determine the interaction between evaluative pressures to perform and formative efforts to support providers, and their potential to lead to desired outcomes.

The supervisor competence includes the ability to promote an effective supervisory relationship, as well as provide useful feedback, which in educational sciences constitutes the difference between summative and formative evaluations [42]. Further, supervisor competence in our data refers to the importance of dedicated, conscientious and respectful supervisors who strive for objectivity and accuracy.

Also, providers and supervisors describe how HC managers may strengthen supervision outcomes through their constructive responses to evaluations, and ability to influence supervisors to assist in solving problems on-site. Also, they may organize internal supervision activities that incorporate results of external supervision. The HC manager is thus an essential actor and facilitator for effective external supervision and its bridge to internal supervision activities.

Both providers and supervisors express a need to train supervisors in how best to support providers within any given pressures to perform, and HC managers appear to need such training as well.

**Fig 2:**
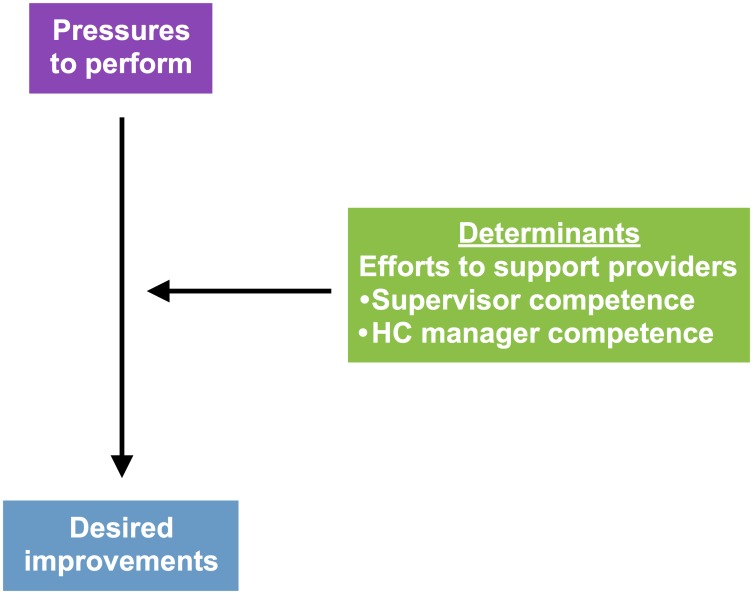
Support as key determinant of the effect of performance pressures.

Fig 1 illustrates these determinants (green box). If the dual role of evaluator and supporter was formally separated into different supervision teams at different times (suggesting the double-arrow would disappear on the level of supervision category) the determining factors would still play a role for each supervision category separately. On the level of supervision activities the double arrow remains, as a system solely operating in a category of evaluative supervision with its adherent performance pressures would be unlikely to yield significant practice improvements without any supportive efforts (see Fig 2).

Fig 1 may be used to analyse the content of any given supervision system (in which other determinants may be present) in the attempt to make underlying power and management processes transparent. We hypothesise most supervision systems have a dominant side either on pressures or support of providers, and that this is determined by whether the supervision system has a managerial main purpose or not. The literature on supervision in healthcare from high-income countries referenced in this paper deals mainly with non-managerial forms emphasising development of provider capacity, such as clinical supervision. Conversely, the referenced publications on supervision from Africa appear as performance- more than provider-oriented. Our impression is that supervisors’ and managers’ competence in relevantly supporting providers will determine how effective a supervision system oriented at performance pressures is in facilitating desired improvements, as illustrated in Fig 2; a modification of Fig 1.

Supervision as a term of multiple applications is elastic. The term managerial supervision points to a normative emphasis. As a set of structure-independent principles the concept of *supportive supervision* seemingly intended to complement or re-invent managerial supervision in Africa [3,13,14]. As such, the dual supervisory role may be the preferred practical alternative to a traditional, non-supportive supervision style, to compensate the pressures within line management systems. But when promises for professional development are given in a supervision system that enforces a PBF-scheme experienced by supervisees as incentive-driven pressures to perform, a strict stance may challenge whether it qualifies for the term supportive, or if that is a euphemism. Naming it supportive may risk masking underlying conflicts related to evaluative objectives [19].

### Emerging challenges

Our data points to a number of challenges when providing support in the context of performance evaluation. There are no indications challenges apply to every supervision encounter, nor that they may not be overcome.

**Fig 3:**
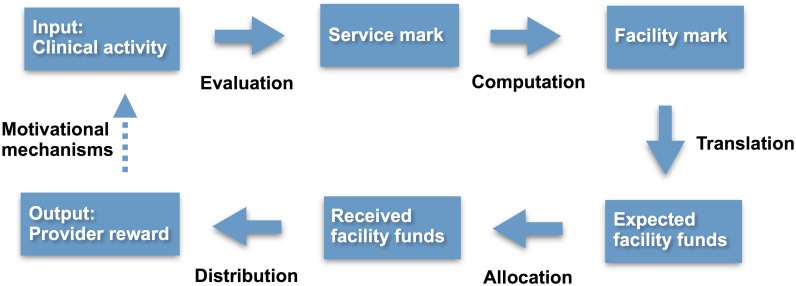
Process from clinical activity to provider reward.

A lack of knowledge among providers about what to expect from supervision, including PBF, is one challenge. Both supervisors and providers expressed providers should know more about the supervision purposes, categories, content and potential consequences. Informational handouts for providers describing categories, activities and responsibilities in supervision could prove helpful [43]. Fig 3 illustrates the steps from work input to PBF output as gathered in our study. To understand how a clinical activity ends as worker rewards one must know how evaluations generate service marks computing into facility marks translating into funds allocated to HCs before a portion of this is distributed as rewards among providers. While this seems incomprehensible, some providers requested insight into at least parts of these processes, in particular evaluation and computation (the latter as indicated by discussions of blame within health facilities).

At the core of PBF are the motivational mechanisms by which provider rewards influence clinical activities (dotted arrow in Fig 3), and further research may elucidate to what extent provider knowledge about these processes may on one hand influence motivation by facilitating a realistic sense of PBF mechanisms, and on the other hand evoke gaming strategies for gain intentions.

Fig 3 also illustrates a fragility of PBF. The motivational mechanism not only depends on clinical input, but also on a fairly perceived allocation and distribution of rewards. Missing allocation or distribution of expected rewards is an actual risk in our data, reported by supervisors to happen. This may be detrimental to a system that built contingency on rewards to regulate motivation. Providers tended to discuss their clinical input and marks rather than the output of rewards when talking about PBF as motivating, which may relate to both above points.

Another challenge was balancing the supervisory roles as evaluator and supporter. As shown in all provider and supervisor discussions, providers tended to perceive supervisors as evaluators, which may indicate that evaluation overshadows other functions. This is a challenge for formative visits as unnecessary fear of evaluation may harm a formative interaction. During evaluations providers may hide themselves or their weaknesses, diminishing the possibility to identify problems and provide grounded feedback and efforts to solve them. Also, we found indications evaluation underpins other supervision functions by linking their content with PBF indicators rather than the problems individual providers experience at work.

One suggestion from supervisors was that monthly evaluation practices (quantité) dictate a disproportionate focus on evaluation, whereas if evaluations were less frequent it may free up time to fulfil provider-oriented formative functions. A recent study in Rwanda showed promising results of an alternative PBF-scheme applying locally developed targets and peer-to-peer learning without depending on regular performance evaluation [44].

Finally, logistic and organizational challenges emerged as important to supervision outcome. Shortage and absence of providers would at times prevent supervision, and lack of transport and preparatory problems e.g. with telecommunication could lead to calling off supervision without warning or to problems with punctuality. All these may lead to mistrust, frustrations and discouragement with supervision. Suggested solutions were highly context-dependent.

### Self-determination theory and motivation

SDT is a theory of motivation vastly utilised in healthcare sciences. It suggests human beings are naturally prone to integrate their experiences into a coherent sense of self to the extent basic needs for relatedness, competence and autonomy are met (see Introduction). External behaviour regulations, such as performance rewards in PBF, may internalize over time to become intrinsic motivation or integrate into individuals’ own values and interests. Primary care providers may have personal values such as accountability, caring and respect [45]. The internalization process is prevented if basic needs are unmet or regulation incompatible with own values. Regulation then remains external or introjects, i.e. operates from a pressured self-demanding, e.g. if self-esteem at work is contingent with good performance evaluations [22,46,47].

Our study almost entirely indicated either external or introjected regulations, the latter expressed e.g. around pressures to perform, and motivation to avoid blame or to attain a feeling of pride related to high marks. External regulation is seen throughout all FGDs as fear of supervision and tendencies to hide from it. At times providers expressed how thriving for high marks may result in better services and learning new skills, indicating at least a potential for an identified (e.g. recognising goals and values of evaluation) or integrated form of regulation.

Several studies question the notion that rewards may diminish (crowd-out) intrinsic motivation [24]. These studies may not differ between introjected and more internalised motivational regulation [22]. Our study showed signs of introjected regulation, suggesting that specification of the level of intrinsic motivation using SDT may be important in studies of the relationship between PBF and intrinsic motivation.

Self-determination theory argues the need for relatedness, competence and autonomy is universal, thus applying to both providers and supervisors. In our study, the need for competence among providers appears insufficiently met considering the abundant complaints of overemphasis on evaluative over formative supervision content. However, it may be that PBF improved providers’ competences compared to before its introduction, inasmuch as increased focus on individual performances may set stronger grounds for internal supervision (from self, peers and HC manager). This is not possible to assess from our study, and may be a research focus in settings where PBF is introduced from anew.

Our study has no indications providers are satisfied with their influence and autonomy at work, but several indications of the opposite. For instance, complaints that poor performance of a colleague in a separate service may influence marks in own services. Or the impression among many supervisors that providers are annoyed with or feel disturbed by supervision. Supervisor autonomy also appeared threatened as some expressed that accountability for performance evaluation leaves little or no time to work as they prefer, i.e. to deliver more formative supervision and support.

The PBF system in Rwanda deliberately avoids employing penalty risks as part of the incentive structure [48]. Yet, providers in our data speak more about risks of low marks, blame and lower income than about chances of rewards. Also, they sometimes engage in blame discussions over who should be punished when certain requirements are not fulfilled. This indicates, as suggested elsewhere [23], that rewards may assume a punitive quality over time.

### Study limitations

External supervision includes both control and support practices, and at study onset it was important to verbalise and discuss our preference towards the latter. Throughout data generation and analysis we also discussed a potential bias among ourselves to sympathise with providers as an underdog in an asymmetric power relation, much like one may sympathise with the patient in a patient-doctor relationship. While these inclinations may have influenced the study, it was our agreed aim to be aware of and discuss them in order to generate data grounded in the reality of supervision, and make interpretations grounded in the data.

The abundance of critical and sensitive information in our data (within a traditionally authoritarian culture where one does not easily criticize another) suggests participants in general felt secure. However, social desirability among colleagues might have triggered exaggerations. We suspect this is not a significant problem, in particular due to several examples where participants disagreed or modified each other.

Our study is an insight into perceptions of 15 providers in 3 of approximately 495 HCs, and 16 supervisors in 3 of 39 districts hospitals at a certain point in time [49]. For transferability to other HCs and districts our triangulation efforts help somewhat as findings were presented to and discussed with providers and supervisors in other regions and district. While our main results were represented across all FGDs we cannot rule out they are exceptions compared to other facilities, although we have no suggestions why they should be. Being a qualitative study of supervision within a context of PBF there are obviously limitations concerning transferability of findings to other countries.

Also, we do not study the development of supervision over time. While PBF incentives are problematized as introjected regulation, the study does not inform whether the locus of causality of motivation was more or less internalized prior to the introduction of PBF.

Our study did not apply systematic observations of supervision as done in other studies [50]. Such could have improved the triangulation process, and presented a possibility to relate perceptions to observed practices.

The perceptions explored in this study may depend on conditions of participants not related to supervision, such as their ability to cover Maslowian needs for food, housing and security based on their work income. E.g. supervision quality is probably the least of worries to nurses who experience several months of salary delay. Our study does not explore such conditions.

Since our study there have been attempts to develop a public system of mentorship at some HCs, of which our study has no explorations.

## Conclusion

In our data the external supervision system was perceived overall as dominated by evaluations for PBF management; a view that could be masked by supervisors’ tendency to accentuate their role as supporters over that as evaluators. Supervisors and providers found formative supervision functions insufficient. The problem seems not the existence of PBF, marks and performance evaluation, but rather their substantial predominance over provider-centered support for professional development. Our study suggests that when supportive or formative supervision is considered secondary or supplementary to a management system, such as in PBF, the quality of support may be at risk, calling for particular strategies to ensure support quality.

Supervisor and provider accounts on supervision quality may differ, as demonstrated in this study. We suggest an essential estimate of supervisory support is accessed through the provider perception, which may also serve as feedback to supervisors.

Using self-determination theory we argue providers introject PBF’s regulatory mechanisms as they push themselves towards higher performance. However, a potential for integrating PBF regulations within own values was indicated. Such internalization seems to require increased competences of supervisors and HC managers to support providers’ in their perceived work challenges, and includes support tailored to address the various forms of pressure providers may be under in a performance-oriented supervision system. Research on how different support strategies may influence motivation in a context of performance pressures is needed, and self-determination theory offers a useful framework.

## Supporting information

S1 Appendix Discussion topics of FGD 1–6. (PDF)

S2 Appendix Study described according to COREQ criteria. (PDF)
