# Supplementary material for: Perceptions on evaluative and formative functions of external supervision of Rwandan primary healthcare facilities: A qualitative study
Source: PLoS One. 2018 Feb 20;13(2):e0189844. doi: 10.1371/journal.pone.0189844 (PMC5819767; doi:10.1371/journal.pone.0189844)
Supplement: S1 Appendix — (PDF) [file pone.0189844.s001.pdf]

S1 Appendix. Discussion topics of FGD 1-6

The 8 discussion topics were written on charts in both Kinyarwanda, English and French, and were presented in the following order:

Discussion topic 1

a) My experience with supervision of health centers [given to supervisors]
b) My experience with supervision from the district hospital [given to providers]

Discussion topic 2

The most important aims of supervision [given to both]

Discussion topic 3

Examples of supervision that went well [given to both]

Discussion topic 4

Examples of supervision that did not go well [given to both]

Discussion topic 5

a) My experiences about supervisees' behaviour [given to supervisors]
b) My experiences about supervisors' behaviour [given to providers]

Discussion topic 6

a) Supervisees' view of our supervision and us [given to supervisors]
b) Supervisors' view of supervision and us [given to providers]

Discussion topic 7

Currently, supervision is mostly composed of clinical training or inspection / evaluation? [given to both]

Discussion topic 8

What should change in the current supervision to improve health services? [given to both]
