# Supplementary material for: Perceptions on evaluative and formative functions of external supervision of Rwandan primary healthcare facilities: A qualitative study
Source: PLoS One. 2018 Feb 20;13(2):e0189844. doi: 10.1371/journal.pone.0189844 (PMC5819767; doi:10.1371/journal.pone.0189844)
Supplement: S2 Appendix — (PDF) [file pone.0189844.s002.pdf]

S2 Appendix. Study described according to COREQ Criteria

Using the COREQ criteria (40) we describe our study in further detail below.

Domain 1: Research team and reflexivity

Personal Characteristics

1. Interviewer/facilitator. Which author/s conducted the interview or focus group?
SI moderated the focus group discussions (FGDs). VC passively listened and observed. MS passively observed and kept a FGD logbook.

2. Credentials. What were the researcher's credentials? E.g. PhD, MD
MS: MD, PhD-student; VC: MD, MMed, PhD-student; SI: BA in social sciences; LN: PhD; PK: MD, PhD, Family Physician.

3. Occupation. What was their occupation at the time of the study?
VC: PhD-student Aarhus University, lecturer by the University of Rwanda, MS: PhD-student Aarhus University, honorary lecturer University of Rwanda; SI: Research assistant; LN: Senior Lecturer, University of Rwanda; PK: Assistant Professor, Aarhus University.

4. Gender. Was the researcher male or female?
VC, MS, SI, PK: male; LN: female. LN did not participate in data gathering.

5. Experience and training. What experience or training did the researcher have?
VC, MS: PhD courses in qualitative research. Teaching about qualitative data analysis at University of Rwanda. LN: PhD within qualitative research, senior lecturer in qualitative research. SI: social science studies including studies in qualitative research. PK: Supervised other qualitative research projects.

Relationship with participants

6. Relationship established. Was a relationship established prior to study commencement?
Among participants a relationship was pre-established with 4 supervisors. These were helpful in helping get contacts according to our sampling strategy. Before a FGD, efforts were done to establish a positive relationship and a comfortable atmosphere as described in paper.

7. Participant knowledge of the interviewer. What did the participants know about the researcher? e.g. personal goals, reasons for doing the research
All participants took part in a presentation round. Researchers presented themselves and their background and role during the FGD. Also, all participants received oral and written information about the purpose of the research. Before commencing a FGD, participants were encouraged to ask any questions, including questions about the researchers and their affiliations. See also paper.

8. Interviewer characteristics. What characteristics were reported about the interviewer/facilitator? e.g. Bias, assumptions, reasons and interests in the research topic.
The FGD moderator (SI) was experienced within social development work, including facilitating meetings and discussions in local communities concerning gender and minority issues. A main interest in this study was to gain further experience with research methods.
VC listened and took notes during FGDs.
MS was the only foreign (white) person present (as observer, keeping a discussion logbook in far background) during all FGDs. A language barrier prevented him from understanding discussions in real-time. Additionally, PK participated as a visiting observer during 2 FGDs to further discuss the method.
Much literature articulates or demonstrates power abuse among supervisors in the African context. MS, VC and SI were particularly acquainted with this as co-authors of a meta-synthesis on supportive supervision in Sub-Saharan Africa(14). This may have led to pre-set sentiments of sympathy with providers and an interest in power aspects. This

bias was discussed throughout planning and data generation. The study design itself was (apart from trying to establish sincere discussions) also a response to this potential bias as an attempt to gather information spontaneously emerging as relevant to informants, and minimize researchers' influence on how topics were discussed.

Domain 2: study design

Theoretical framework

9. Methodological orientation and Theory. What methodological orientation was stated to underpin the study? e.g. grounded theory, discourse analysis, ethnography, phenomenology, content analysis.
We do not claim the study belongs to a particular theoretical or methodological tradition. Like Miles, Huberman and Saldana (in "Qualitative Data Analysis, A methods Sourcebook", 3rd edition 2014) we consider ourselves *pragmatic realists*, in that we regard qualitative data as generated through the interaction of researchers and participants, and reflects both parties ideas about the world, yet that social phenomena are more than just constructions in the minds of people, but also exist with some degree of stability in the world allowing it to be observed, described and interpreted. In other words, we believe there is a social reality to be described, although its description may vary depending on the social actors (and researchers) observing. This led us to a pragmatic methodology aiming to minimize researcher-desirability among participants. We consider our data to reflect narratives of people, and do not take them as precise nor necessarily truthful accounts. Our analysis process is best described as inductive, thematic analysis using systematic open coding. The process is given in further detail in the paper.

Participant selection

10. Sampling. How were participants selected? e.g. purposive, convenience, consecutive, snowball
Purposive sampling. Please see details in paper.

11. Method of approach. How were participants approached? e.g. face-to-face, telephone, mail, email
For supervisors, the head of the supervising team was telephoned and asked to gather his team for the discussion. For providers, the titulaire of the HC was contacted.

12. Sample size. How many participants were in the study?
31 participants for FGDs, see table 1. Several other supervisors and providers provided informal feedback and insights. During meetings to present and discuss the findings an additional 10 new supervisors and 8 new providers participated.

13. Non-participation. How many people refused to participate or dropped out? Reasons?
None of those asked to participate refused.

Setting

14. Setting of data collection. Where was the data collected? e.g. home, clinic, workplace
All data was collected at the participants' own workplace, that is for providers at their health centre, and for supervisors at the district hospital. Health centre managers decided the best time for conducting FGDs with providers, in all cases in the afternoon after clinical duties.

15. Presence of non-participants. Was anyone else present besides the participants and researchers?
No one beyond participants and researchers were present during the discussions.

16. Description of sample. What are the important characteristics of the sample? e.g. demographic data, date
Please see paper and table 1. Time period of FGDs also specified in paper.

Data collection

17. Interview guide. Were questions, prompts, guides provided by the authors? Was it pilot tested?
Discussion guide was pilot tested. After each discussion, results were discussed among 3 researchers, including if changes were needed in the guide. See supplementary material 1 for the discussion topics. A full discussion guide may be provided upon request.

18. Repeat interviews. Were repeat interviews carried out? If yes, how many?
No. But a mixed FGD presenting excerpts from other discussions was conducted with 2 supervisors and 3 providers. See paper for details. Also, as mentioned 3 meetings were held with a total of 18 new participants to present, refine and discuss findings and interpretation.

19. Audio/visual recording. Did the research use audio or visual recording to collect the data?
All discussions were audio recorded.

20. Field notes. Were field notes made during and/or after the interview or focus group?
Field notes done both during and after discussions. Also, a group reflection was done with 3 researchers after each discussion using field notes. Non-systematic field notes were taken during observations of supervision visits.

21. Duration. What was the duration of the interviews or focus group?
Please see the paper.

22. Data saturation. Was data saturation discussed?
Data saturation is a debated phenomenon. A perception of saturation may stem from researchers' limitations in asking good questions or designing an optimum environment for gaining new information. We sensed several repetitions of themes in our data already after 4 FGDs (2 in each staff group). After 6 FGDs (3 in each group) the material was transcribed and translated, and discussed among 3 researchers. We found an abundance of commonalities between FGDs of the same staff group (supervisors and providers), and concerning our research questions few (largely insignificant) themes were unique to a single FGD. We therefore find it fair to claim our data is sufficiently saturated in terms of our study focus.

23. Transcripts returned. Were transcripts returned to participants for comment and/or correction?
Transcripts were not returned to participants for their comment or correction. All transcripts and translations were control checked in toto by the discussion moderator.

Domain 3: analysis and findings

Data analysis

24. Number of data coders. How many data coders coded the data?
4 people did inductive coding, and discussed the structure of a final harmonised code tree. 2 coders applied this code tree, which was harmonized to one final coding. Codes were applied to whole paragraphs, or in cases with long paragraphs to a part of it.

25. Description of the coding tree. Did authors provide a description of the coding tree?
See main text for coding procedure. The code tree contains a main section related to the supervisory relationship, which will be utilized in a separate study, and another section related to the institution of supervision, including purposes, structure, process, competences and motivation, as discussed in this paper. There were several other codes, such as for indications of group dynamic. A code tree may be provided upon request.

26. Derivation of themes. Were themes identified in advance or derived from the data?
Themes were not identified in advance, and were for most cases different from the discussion topics. They reflect an inductive coding process. They represent for this paper, as mentioned, 4 conceptual levels in which evaluative and formative supervision functions were discussed. We used the same FGD approach in all discussions.

27. Software. What software, if applicable, was used to manage the data?
MAXQDA 11. During the open coding among 4 coders other programs were testet.

28. Participant checking. Did participants provide feedback on the findings?
The final manuscript was presented for feedback to one participating English-speaking supervisor, who had provided feedback to questions throughout the entire study period. A group of participating supervisors joined a presentation

of our findings and provided feedback. Several non-participating providers and supervisors also were presented our findings for the sake of feedback and discussion (as reported in the paper).

Our data has been nuanced through a number of triangulation sources as listed in S2 Appendix Table 1, below:

Table 1. Characteristics of triangulation sources

Source	Specification	Number	Purpose
**Participant-observations***	MS participation in external supervision visit	6 visits	Gain direct sense of supervision environment and supervisory interaction
	MS and VC visits to health centres in various districts	> 20 visits	Understand work conditions at health centres
**Informal conversations***	With two key informant supervisors	5 meetings. Numerous telephone and email conversations	Discuss formal supervision structure and content. Usually to clarify questions emerging from observations or FGDs
	With providers	10 individual providers	Explore supervision views of providers who did not participate in FGDs, as a further exploration of perceptions
	With health centre managers	4 health centre managers	Check health centre managers' view of main findings to not miss potential disagreements
**Review of written material***	Local assistant (SI) reviewed internal supervision guidelines and tools provided by key informants	13 document files, 3 excel files, 1 power point presentation (majority in French or Kinyarwanda)	Understand specific content of supervision through supervision tools, with special focus on the described role of formative functions.
**Presenting FGD findings to participants****	Supervisor meeting	Meeting with 7 supervisors, 3 of whom participated in FGDs	Ensure acceptable interpretation of data
	Provider meeting	Informal conversations with 2 providers from 1 FGDs	Ensure acceptable interpretation of data
**Presenting FGD findings to non-participants ****	Supervisor meeting	6 supervisors in region other than those for data generation	Check transferability and acceptability of findings among non-involved sites. Nuance findings.
	Provider meeting	8 providers in region other than those for data generation	Check transferability and acceptability of findings among non-involved sites. Nuance findings.

FGDs: Focus group discussions; * Before, during and after FGDs; ** After FGDs.

Reporting

29. Quotations presented. Were participant quotations presented to illustrate the themes/findings? Was each quotation identified? e.g. participant number

Quotations make up the main method of presenting results. This was because we found numerous illustrative quotations, that in our view describe our study findings better than we could have done with paraphrases alone. For each quote we present the professional group (supervisor or provider), gender and for providers also the nursing degree. We did not use participant numbers as ID as we wanted to eliminate any potential risk of identification, such

as supervisor participants being able to identify a provider participant from their own district based on a sum of quotations. An omitted intermediate section is shown as (…). Sections found to change or bear significant meaning in a quote were not omitted.

30. Data and findings consistent. Was there consistency between the data presented and the findings?
We believe the data presented through quotes are grounded in and are a fair representation of all transcripts. A number of efforts, as described, were done to confirm our findings including several meetings with providers and supervisors.

31. Clarity of major themes. Were major themes clearly presented in the findings?
Major themes of relevance to this paper have been presented. There were other major themes concerning the supervisory relationship, which will be reported in a separate paper. Finally, several themes were not found relevant to any of the papers, such as issues concerning logistic and time management. Also, several themes about worker shortage and other problems concerning the health workforce have not been presented. This article only focuses on issues pertaining to the formative and evaluative functions of supervision in Rwanda.

32. Clarity of minor themes. Is there a description of diverse cases or discussion of minor themes?
Certain minor themes are presented within major themes in the result section, as well as discussed in the discussion section.
